# Early trajectories of skin thickening are associated with severity and mortality in systemic sclerosis

**DOI:** 10.1186/s13075-020-2113-6

**Published:** 2020-02-18

**Authors:** Emmanuel Ledoult, David Launay, Hélène Béhal, Luc Mouthon, Grégory Pugnet, Jean-Christophe Lega, Christian Agard, Yannick Allanore, Patrick Jego, Anne-Laure Fauchais, Jean-Robert Harlé, Sabine Berthier, Achille Aouba, Arsène Mekinian, Elisabeth Diot, Marie-Elise Truchetet, Carine Boulon, Alain Duhamel, Eric Hachulla, Vincent Sobanski, the French National Scleroderma Cohort Network, Zahir Amoura, Olivier Aumaitre, Eric Auxenfants, Marie-Hélène Balquet, Cristina Belizna, Boris Bienvenu, Emmanuel Chatelus, Robin Dhote, Yves Frances, Jean-Baptiste Gaultier, Bernard Imbert, Jean-Emmanuel Kahn, Gilles Kaplanski, Pierre Kieffer, Noémie Le Gouellec, Philippe Guilpain, Olivier Lidove, Nadine Magy-Bertrand, François Maurier, Thomas Papo, Jean-Loup Pennaforte, Jacques Pouchot, Vivianne Queyrel, Denis Wahl

**Affiliations:** 1 Univ. Lille, Institute for Translational Research in Inflammation (INFINITE), F-59000 Lille, France; 2 CHU Lille, Service de Médecine Interne, Centre de Référence des Maladies Auto-immunes et Systémiques Rares du Nord et Nord-Ouest de France (CeRAINO), F-59000 Lille, France; 3 INSERM, U1286, F-59000 Lille, France; 4 Univ. Lille, CHU Lille, EA 2694-Santé publique, épidémiologie et qualité des soins, Unité de Biostatistiques, F-59000 Lille, France; 5 Hôpital Cochin–APHP, Service de Médecine Interne, Paris, France; 6 CHU Toulouse, Service de Médecine Interne, Toulouse, France; 7 CHU Lyon Sud, Service de Médecine Interne, Pierre-Bénite, France; 8 CHU Nantes, Service de Médecine Interne, Nantes, France; 9 Hôpital Cochin–APHP, Service de Rhumatologie, Paris, France; 10 CHU Rennes, Service de Médecine Interne, Rennes, France; 11 CHU Limoges, Service de Médecine Interne, Limoges, France; 12 Hôpital de la Timone, Service de Médecine Interne, Marseille, France; 13 CHU Dijon, Service de Médecine Interne et Immunologie Clinique, Dijon, France; 14 CHU Caen, Service de Médecine Interne, Caen, France; 15 Hôpital Saint-Antoine–APHP, Service de Médecine Interne, Paris, France; 16 CHU Tours, Service de Médecine Interne, Tours, France; 17 CHU Bordeaux, Service de Rhumatologie, Bordeaux, France; 18 CHU Bordeaux, Service de Médecine vasculaire, Bordeaux, France

**Keywords:** Systemic sclerosis, Modified Rodnan skin score, Skin thickening trajectories, Clinical heterogeneity

## Abstract

**Background:**

Systemic sclerosis (SSc) is a severe and highly heterogeneous disease. The modified Rodnan skin score (mRSS) is a widely used tool for the assessment of the extent and degree of skin thickness. This study aimed to identify the classes of patients with early similar skin thickening trajectories without any a priori assumptions and study their associations with organ involvement and survival.

**Methods:**

From the French SSc national cohort, patients with a disease duration of less than 2 years at inclusion and with at least 2 mRSS available within the first 4 years of follow-up were enrolled. Classes of patients with similar mRSS trajectories were identified based on a latent class mixed model. The clinical characteristics and survival rate were compared between the obtained classes.

**Results:**

A total of 198 patients fulfilled the inclusion criteria, with a total of 641 mRSS available. The median disease duration and follow-up were 0.8 (interquartile range 0.4; 1.2) and 6.3 (3.8; 8.9) years, respectively. Individual trajectories of mRSS were highly heterogeneous between patients. Models with 1–6 latent classes of trajectories were sequentially assessed, and the 5-class model represented the best fit to data. Each class was characterized by a unique global trajectory of mRSS. The median disease duration did not differ significantly between classes. Baseline organ involvement was more frequent in classes with significant change over time (classes 2–5) than in class 1 (low baseline mRSS without significant change over time). Using Cox regression, we observed a progressively increasing risk of death from classes 1 to 5.

**Conclusions:**

Early identification of clinical phenotype based on skin thickening trajectories could predict morbi-mortality in SSc. This study suggested that mRSS trajectories characterization might be pivotal for clinical practice and future trial designs.

## Background

Systemic sclerosis (SSc) is a chronic connective tissue disease characterized by widespread fibrosis of the skin and/or internal organs [1]. Among the hallmarks of SSc, skin thickening is one of the pivotal symptoms and is used in routine practice to classify patients within the subsets—limited cutaneous SSc (lcSSc) and diffuse cutaneous SSc (dcSSc) [2]. Modified Rodnan skin score (mRSS) is a semiquantitative score, ranging from 0 (normal) to 3 (severe), used to evaluate the skin thickness in 17 different cutaneous sites (for a total score from 0 to 51), and is correlated with histological skin thickness [3]. mRSS is a validated clinical instrument [4] often used as primary or secondary outcomes in clinical trials [5].

Few studies have focused on the evolution of mRSS over time. Using latent trajectory modeling, Shand et al. [6] divided the early dcSSc patients (less than 2 years after disease onset) into three subgroups: “low baseline/improvers,” “high baseline/improvers,” and “high baseline/non-improvers.” Survival was associated with the subsequent mRSS trajectories, albeit only 68% of patients included could be included in one of these 3 groups. Perera et al. [7] described five skin thickening profiles in anti-topoisomerase I antibodies positive SSc patients early in the course of the disease. The 3 subgroups of dcSSc patients based on their skin thickening progression rate (STPR) were as follows: rapid (≥ 40 units per year), intermediate (15–40 units per year), and slow (≤ 15 units per year). The two subgroups of lcSSc patients were as follows: one where the skin clinical phenotype subsequently became diffuse, and the remaining one was limited throughout the follow-up. These studies underlined the well-known heterogeneity of the skin thickening evolution in SSc, and the complexity of the relations between mRSS at baseline and the skin thickening course (improvers/non-improvers and STPR). Some predictive factors of mRSS progression (defined as > 5 units and ≥ 25% increment in mRSS at 1 year follow-up) have been identified such as tendon friction rubs, joint synovitis, mRSS at baseline ≤ 22/51, disease duration < 15 months, and antibody status [8–10]. Nevertheless, it remains difficult to accurately predict the trajectory of mRSS in a given patient, which might limit the homogeneity and thus comparability of patients included in clinical trials [10]. Deciphering the skin thickening heterogeneity is therefore of utmost importance considering the wide use of mRSS as the primary outcome and the three recent negative clinical trials to prove a benefice on skin thickening in SSc [11–13]. We herein aimed to identify the early mRSS longitudinal trajectories in SSc patients from the prospective French SSc national database without any a priori assumptions and to examine their associations with organ involvement and survival.

## Patients and methods

### Study population and inclusion criteria

The French SSc national database is a multicenter observational study conducted in 42 French hospital centers. Adult SSc patients under standard care were enrolled consecutively since 2010. Data were retrospectively collected before 2010 and then prospectively collected using a standardized form recorded on an online database (Clean Web®) with plausibility checks. In accordance with the French legislation, the database has received ethical approval from CCTIRS (approval no. 13.145; Advisory Committee on Information Processing in Material Research in the Field of Health). Data protection complied with the requirements of the National Information science and Liberties Commission and recorded under no. 914607. All included patients provided informed consent. Patients could also be included in other cohorts (e.g., European Scleroderma Trials and Research group) in a non-competitive way. Data were extracted in August 2015, and patients were eligible for the present analysis if (i) the ACR 1980 preliminary classification criteria [14] and/or 2013-ACR/EULAR SSc classification criteria [15] were fulfilled, (ii) inclusion visit occurred less than 2 years after the first onset of non-Raynaud phenomenon (RP) symptom, and (iii) the baseline mRSS and at least 1 mRSS during follow-up were available.

### Data collection and variables

Baseline was defined as the date of inclusion in the database and follow-up as the time between the inclusion and the last available visit at the time of the extraction. Data on demographics, dates of first RP and first non-RP symptom, cutaneous subset, telangiectasia, calcinosis, and autoantibody status (anti-centromere [ACA], anti-topoisomerase I [ATA], anti-RNA polymerase III [anti-RNAP3], anti-U1 RNP, anti-PM/Scl, and other autoantibodies) were collected. Disease duration was defined as the time between the first non-RP symptom by patient report and inclusion visit. Organ involvements were defined by the occurrence of clinical events at baseline—skin involvement: mRSS and STPR defined as the mRSS at baseline visit divided by the disease duration (in years) [7, 16]; joint involvement: arthritis, arthralgia, friction rubs, or synovitis; muscle involvement: myalgia, myositis, or rhabdomyolysis; lung involvement: interstitial lung disease (ILD) diagnosed on high-resolution computerized tomography or chest X-ray, forced vital capacity (FVC % predicted value), and diffusing capacity of the lung for carbon monoxide (DLCO % predicted value); heart involvement: arrhythmia or conduction block or systolic dysfunction (left ventricular fraction ejection ≤ 45% of predicted value) or pericardial effusion; pulmonary hypertension (PH): mean pulmonary arterial pressure measured by right heart catheterization > 25 mmHg at rest; gastrointestinal tract (GIT) involvement: esophageal reflux, dysmotility, constipation, diarrhea, signs of bacterial overgrowth and/or malabsorption, abnormal esophageal manometry, and/or endoscopy test; digital ulcer (DU): history or active DU, digital tip, pitting scar, or digital ischemia; and scleroderma renal crisis (SRC): defined by new onset hypertension (≥ 150/85 mmHg) associated with a decrease in renal function defined by a decrement of at least 10% in the estimated glomerular filtration rate. C-reactive protein elevation was defined as a C-reactive protein level of > 6 mg/L. All immunosuppressive drugs were recorded during the follow-up. Death was also recorded.

### Statistical analyses

The primary objective of this study was to delineate groups of patients according to their skin thickening trajectories (classes) as measured by mRSS over time using latent class mixed models (LCMM) [17, 18]. LCMM assumes that the population is divided into a finite number of groups called latent classes. Each latent class is characterized by a specific mean trajectory, which is described by a class-specific linear mixed model. In a given latent class, the individual trajectories of patients are close to each other, while the individual trajectories of patients of different classes tend to be dissimilar. In LCMM, latent classes correspond to an unknown categorical variable which is identified from data using a multinomial logistic model, and trajectories of mRSS are analyzed using the mixed model with random coefficients to take into account individual trajectories. The random effects (linear or quadratic) are determined from the analysis of residuals according to Verbeke and Molenberghs [18]. To identify the number of classes, several LCMMs are performed. Each model predicts the shape of the trajectory of each class, estimates the probability for each individual of class membership and assigns each of them to the class for which the likelihood is the highest. Time 0 was defined by the date of baseline mRSS recorded. Trajectories were censored after 4 years of follow-up because of substantial missing records after this duration. To determine the best number of latent classes that represented the heterogeneity of developmental trajectories, we considered both formal statistical criteria (such as Bayes information criterion (BIC)) and model adequacy. A low value of BIC and average posterior probabilities of class membership greater than 0.7–0.8 correspond to the better model [19, 20]. A sensitivity analysis was performed with disease duration as an adjustment factor in the different LCMM.

Continuous variables were expressed as mean ± standard deviation (SD) or median and interquartile range (IQR) to describe classes, and as mean with 95% confidence interval (CI) to describe trajectory shapes. The normality of distribution was checked graphically and using the Shapiro-Wilk test. Categorical variables were expressed as frequencies and percentages. Comparisons of classes were performed using the analysis of variance or the Kruskal-Wallis test for quantitative variables and the Fisher’s exact test or the chi-square test for categorical variables. In the case of significant results, pairwise comparisons were performed, and a Bonferroni correction was applied. The survival rate in every class was estimated using the Kaplan-Meier method and compared using the Cox regression adjusted for age and sex. All statistical tests were performed at 2-tailed α level of 0.05. All data analyses were performed using the SAS software version 9.4 (SAS Institute Inc., Cary, NC, USA) and the R software (Package LCMM).

## Results

### Baseline characteristics

**Fig. 1:**
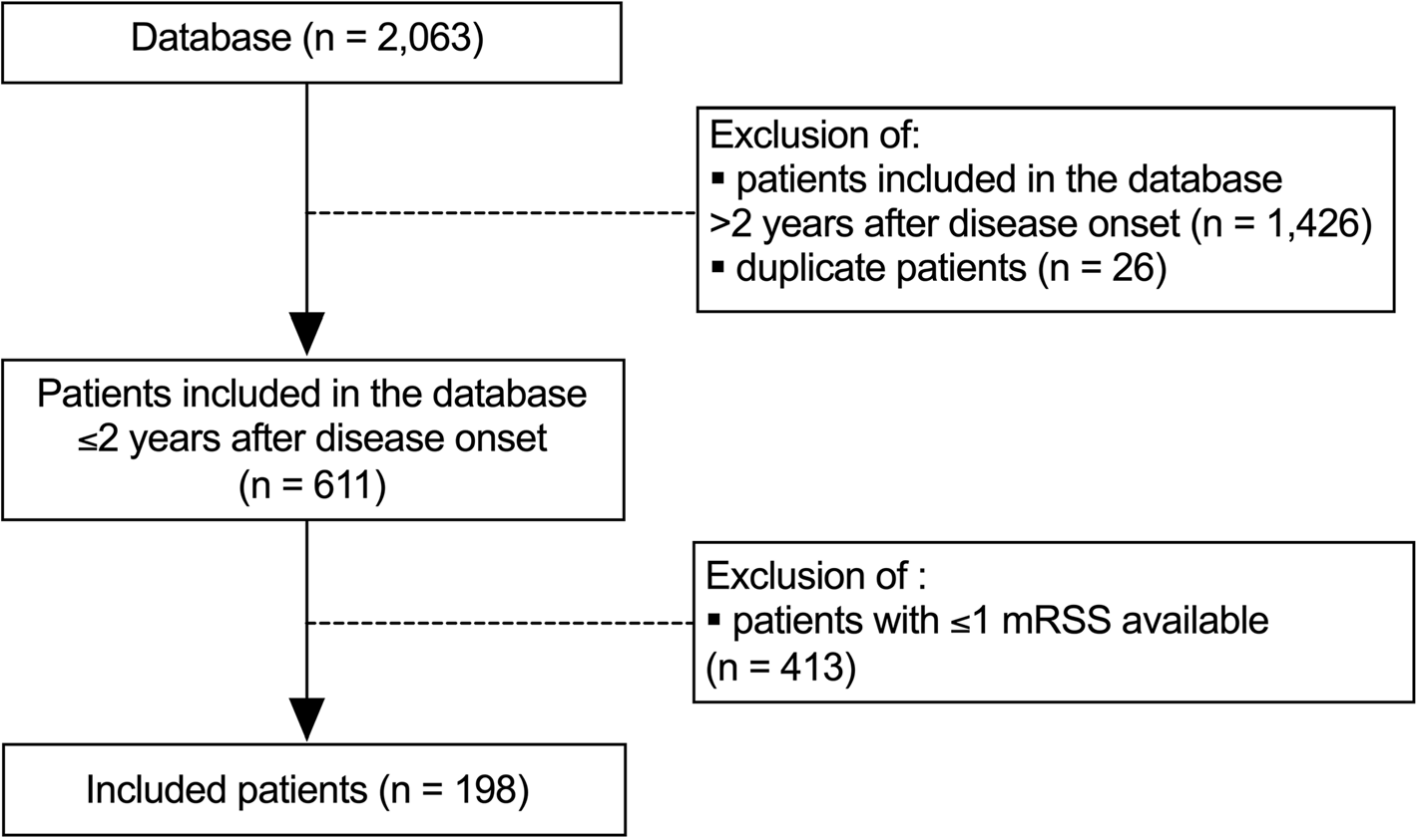
Flow-chart*.* RP: Raynaud’s phenomenon; mRSS: modified Rodnan skin score

Of the 2063 patients in the database, 611 had a disease duration of less than 2 years at the time of inclusion (median disease duration (IQR): 0.7 (0.3; 1.2) years). Among them, 198 patients with an available baseline mRSS and at least 1 mRSS obtained within the first 4 years of follow-up were included (Fig. 1). The mean age of included patients was 51.1 ± 14.3 years, and majority of them were White patients; the male to female ratio was 3:1. The median disease duration at baseline and follow-up were 0.8 (IQR 0.4; 1.2) years and 6.3 (3.8; 8.9) years, respectively. The proportion of dcSSc patients was 49.7%. Nearly 95.0% of patients were positive for antinuclear antibodies (ACA 28.3%, ATA 55.9%, and anti-RNAP3 5.3%). The median baseline mRSS was 8 (2; 18) (Table 1). The proportion of male patients with dcSSc, ATA, and anti-RNAP3 was higher in the group included than in those with ≤ 1 mRSS (Additional file 1). No significant difference was observed in the mortality rate between the 2 groups (log-rank test, *p* = 0.40). A total of 641 mRSS values were available, and 55.0% of the patients had at least 3 mRSS records (Additional file 2).

**Table 1:** Demographics and disease characteristics of included patients (*n* = 198)

	No. with available data	Included patients (*n* = 198)
Demographics		
Sex, female, no. (%)	198	145 (73.2)
Ethnicity, no. (%)		
White	161	140 (87.0)
Black	161	16 (9.9)
Asian	161	5 (3.1)
Age, mean ± SD, years	198	51.1 ± 14.3
Diseases characteristics		
Autoantibody status ^a^, no. (%)		
Anti-nuclear antibody positive	165	156 (94.5)
Anti-centromere	152	43 (28.3)
Anti-topoisomerase	152	85 (55.9)
Anti-RNAP3	152	8 (5.3)
Anti-U1 RNP	152	9 (5.9)
Anti-PM/Scl	152	6 (4.0)
Disease duration, median (IQR), years	198	0.8 (0.4; 1.2)
Duration from RP, median (IQR), years	187	1.3 (0.6; 3.9)
Follow-up, median (IQR), years	198	6.3 (3.8; 8.9)
Skin variables		
Cutaneous subset, limited, no. (%)	195	98 (50.3)
mRSS, baseline, median (IQR)	198	8 (2; 18)
Baseline organ involvement, no. (%)		
Telangiectasia	183	76 (41.5)
Calcinosis	175	20 (11.4)
Joints	191	114 (59.7)
Muscles	194	53 (27.3)
Digital ulcers	181	76 (42.0)
Gastrointestinal tracts	187	99 (52.9)
Interstitial lung disease	181	72 (39.8)
FVC, median % (IQR)	160	96.0 (76.0; 108.0)
DLCO, median % (IQR)	155	63.0 (50.0; 78.0)
Heart	187	15 (8.0)
Pulmonary hypertension	194	15 (7.7)
Renal crisis	123	12 (9.8)
Biological variable, no. (%)		
Baseline CRP level, ≥ 6 mg/L	148	51 (34.5)
Treatments ^b^, no. (%)		
Steroids and/or IS	189	127 (68.7)

Numbers are given as % or mean ± standard deviation (SD) or median with interquartile range. *Anti-RNAP3* anti-RNA polymerase III antibodies, *CRP* C-reactive protein; *disease duration* duration from the first non-RP symptom, *DLCO* diffusing capacity of the lung for carbon monoxide (% predicted value), *FVC* forced vital capacity (% predicted value), *RP* Raynaud’s phenomenon

^a^The sum of % may be different from 100% because some patients had either unidentified ANA or multiple autoantibodies

^b^During follow-up

### Model fit evaluation

**Fig. 2:**
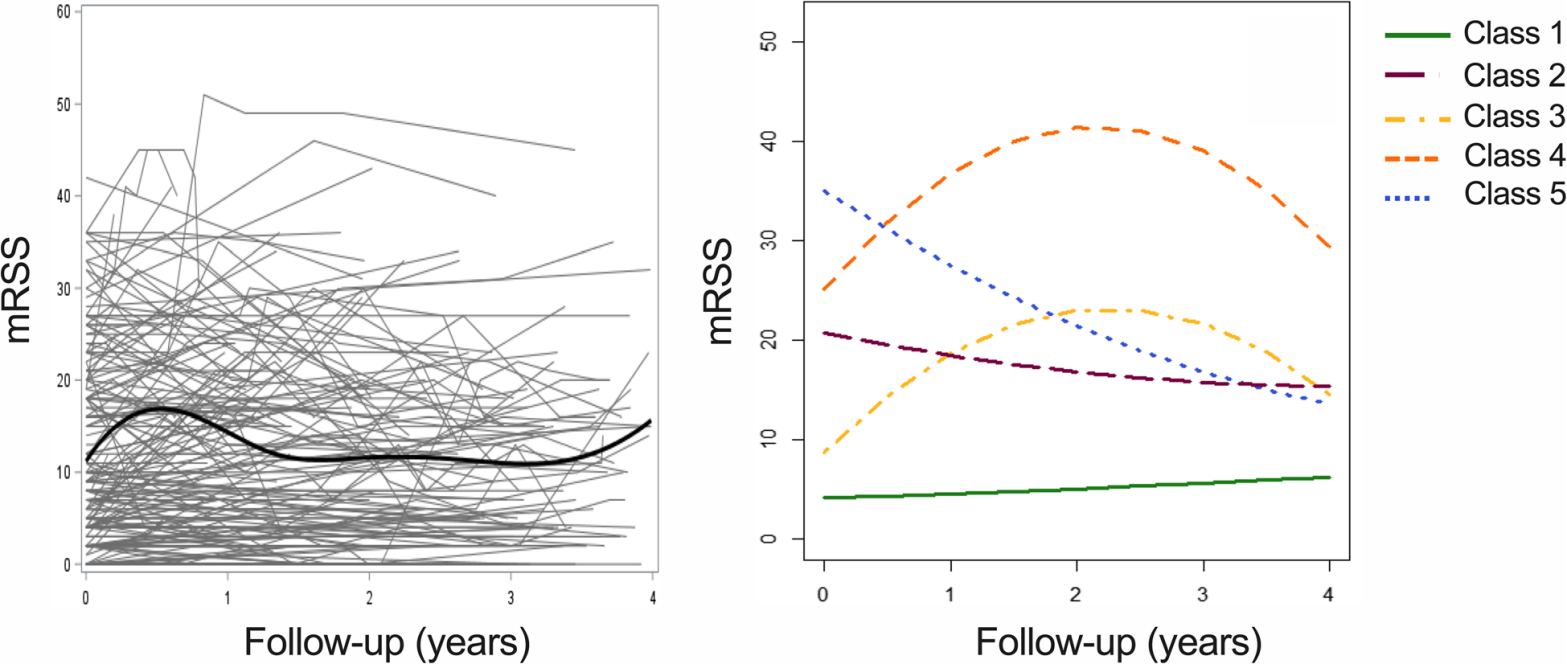
The 5-class LCMM results*.* (Left) All individual trajectories and the average trend estimated using B-splines. (Right) Results of the 5-class LCMM. Time 0 was defined by the date of baseline mRSS record. mRSS: modified Rodnan skin score

Individual trajectories of 198 patients included are presented in Fig. 2 and showed a notable heterogeneity between patients. Models with 1 to 6 latent classes were sequentially performed (Additional file 3). The 5-class model had the lowest value of BIC index, which suggests that it represented the best fit to data (Fig. 2, Additional file 4). The averages of posterior probabilities of belonging to a class (indicating that the modeled trajectories gathered individuals with similar patterns of skin change and distinguished the aforementioned individuals from those with dissimilar patterns of skin change) were 0.96, 0.88, 0.92, 0.95, and 0.93, respectively, for classes 1 to 5 (Additional file 5, Additional file 6). The median disease duration did not differ significantly between classes (*p* = 0.21). A sensitivity analysis with disease duration as an adjustment factor yielded similar results and confirmed that the 5-class model best fitted the data (Additional files 7, 8, and 9).

### Demographics and clinical characteristics of the 5 mRSS trajectory classes (Table 2, Fig. 3, Additional file 10)

*Class 1* was characterized by a low baseline mRSS (mean mRSS 4.1 [95% CI 3.2; 5.0]) with no significant change over time (mean mRSS at 1 year, 2 years, 3 years, and 4 years: 4.6 [3.3; 5.9], 5.1 [3.7; 6.4], 5.6 [4.3; 6.9], and 6.2 [3.8; 8.6], respectively). This class included 117 patients with lcSSc (82.6%), which primarily affects White women with ACA (42.3%) or ATA (42.3%). Almost all ACA-positive patients (95%) were assigned to this class. At baseline, one-third of the patients had ILD, joint, GIT, and DU involvements. The median STPR was 3.9 (IQR 1.2; 9.3) units/year.

**Table 2:** Demographics and disease characteristics of the 5 mRSS trajectories classes (*n* = 198)

	No. with available data	Class 1 (*n* = 117)	Class 2 (*n* = 43)	Class 3 (*n* = 13)	Class 4 (*n* = 13)	Class 5 (*n* = 12)	*p*
Demographics							
Sex, female, no. (%)	198	93/117 (79.5)	28/43 (65.1)	9/13 (69.2)	7/13 (53.9)	8/12 (66.7)	0.13
Ethnicity, no. (%)							<.001
White	161	88/98 (89.8)	28/32 (87.5)	8/11 (72.7)	8/11 (72.7)	8/9 (88.9)	
Black	161	6/98 (6.1)	3/32 (9.4)	3/11 (27.3)	3/11 (27.3)	1/9 (11.1)	
Asian	161	4/98 (4.1)	1/32 (3.1)	0/11 (0.0)	0/11 (0.0)	0/9 (0.0)	
Age, mean ± SD, years	198	52.9 ± 13.9	49.2 ± 14.1	43.6 ± 17.0	47.8 ± 14.6	51.9 ± 14.5	0.15
Disease characteristics							
Disease duration, median (IQR), years	198	0.8 (0.4; 1.3)	0.9 (0.5; 1.2)	0.9 (0.5; 1.1)	0.6 (0.2; 0.9)	1.1 (0.4; 1.3)	0.21
Duration from RP, median (IQR), years	187	1.6 (0.6; 6.0)	1.3 (0.6; 1.7)	0.8 (0.8; 2.0)	0.6 (0.2; 0.9)	1.3 (0.4; 3.3)	0.018
Follow-up, mean ± SD, years	198	4.9 ± 0.2	5.0 ± 0.6	6.8 ± 1.0	7.4 ± 1.5	4.1 ± 0.4	0.10
Antibodies ^a^, no. (%)							
Anti-nuclear	165	89/93 (95.7)	37/39 (94.8)	9/11 (81.8)	11/12 (91.7)	10/10 (100)	0.32
Anti-centromere	152	41/97 (42.3)	1/28 (3.6)	0/9 (0.0)	1/10 (10.0)	0/8 (0.0)	<.001
Anti-topoisomerase I	152	41/97 (42.3)	21/28 (75.0)	8/9 (88.9)	8/10 (80.0)	7/8 (87.5)	<.001
Anti-RNAP3	152	3/97 (3.1)	4/28 (14.3)	0/9 (0.0)	0/10 (0.0)	1/8 (12.5)	0.11
Anti-U1RNP	152	7/97 (7.2)	2/28 (7.1)	0/9 (0.0)	0/10 (0.0)	0/8 (0.0)	NA
Anti-PM/Scl	152	5/97 (5.2)	1/28 (3.6)	0/9 (0.0)	0/10 (0.0)	0/8 (0.0)	NA
Only others	152	4/97 (4.1)	1/28 (3.6)	1/9 (11.1)	2/8 (20.0)	0/8 (0.0)	0.19
Skin variables							
Cutaneous subset, limited, no (%)	195	95/115 (82.6)	1/42 (2.4)	2/13 (15.4)	0/13 (0.0)	0/12 (0.0)	<.001
STPR, median (IQR); units per years	198	3.9 (1.2; 9.3)	21.8 (16.0; 35.9)	7.8 (5.7; 13.4)	38.5 (29.0; 131.3)	34.4 (27.6; 74.9)	<.001
mRSS trajectories, [95% CI]							
Mean baseline mRSS	198	4.1 [3.2; 5.0]	20.8 [19.0; 22.5]	8.7 [6.0; 11.5]	25.1 [22.6; 27.6]	35.1 [32.2; 37.9]	NA
Mean mRSS at 6 months	198	4.3 [3.3; 5.4]	19.5 [17.7; 21.3]	14.5 [11.4; 17.6]	31.9 [28.7; 35.1]	31.1 [27.6; 34.6]	NA
Mean mRSS at 1 year	198	4.6 [3.3; 5.9]	18.4 [16.3; 20.5]	18.7 [14.8; 22.7]	36.9 [32.7; 41.0]	27.6 [23.3; 31.8]	NA
Mean mRSS at 2 years	198	5.1 [3.7; 6.4]	16.8 [14.5; 19.0]	23.1 [18.5; 27.6]	41.5 [37.0; 46.1]	21.5 [17.2; 25.7]	NA
Mean mRSS at 3 years	198	5.6 [4.3; 6.9]	15.7 [13.6; 17.9]	21.6 [17.8; 25.5]	39.1 [35.3; 42.8]	16.8 [10.1; 23.5]	NA
Mean mRSS at 4 years	198	6.2 [3.8; 8.6]	15.4 [11.0; 19.8]	14.5 [8.4; 20.7]	29.5 [22.7; 36.2]	13.5 [0; 29.5]	NA
Baseline organ involvement, no. (%)							
Telangiectasia	183	48/109 (44.0)	15/38 (39.5)	4/12 (33.3)	4/13 (30.8)	5/11 (45.5)	0.85
Calcinosis	175	13/104 (12.5)	3/37 (8.1)	2/11 (18.2)	2/12 (16.7)	0/11 (0.0)	0.57
Joints	191	53/114 (46.5)	31/40 (77.5)	11/13 (84.6)	10/13 (76.9)	9/11 (81.8)	<.001
Muscles	194	20/117 (17.1)	19/40 (47.5)	5/13 (38.5)	5/13 (38.5)	4/11 (36.4)	0.001
Digital ulcers	181	33/111 (29.7)	19/34 (55.9)	9/13 (69.2)	6/12 (50.0)	9/11 (81.8)	<.001
Gastrointestinal tracts	187	51/111 (46.0)	25/40 (62.5)	9/12 (75.0)	9/12 (75.0)	5/12 (41.7)	0.062
Interstitial lung disease	181	34/106 (32.1)	19/40 (47.5)	8/12 (66.7)	7/11 (63.6)	4/12 (33.3)	0.040
FVC, median % (IQR)	160	101 (86.0; 111.0)	86.0 (70.8; 102.5)	88.5 (75;0; 111.5)	88.0 (68.5; 93.0)	82.0 (55.0; 102.0)	0.009
DLCO, median % (IQR)	155	66.0 (50.0; 80.0)	56 (43.0; 69.0)	67.0 (53.0; 72.0)	65.0 (47.0; 86.9)	61.0 (42.0; 69.0)	0.35
Heart	187	7/109 (6.4)	3/41 (7.3)	2/13 (15.4)	2/12 (16.7)	1/12 (8.3)	0.40
Pulmonary hypertension	194	10/114 (8.8)	2/42 (4.8)	1/13 (7.7)	2/13 (15.4)	0/12 (0.0)	0.58
Renal crisis	123	1/59 (1.7)	7/31 (22.6)	1/11 (9.1)	3/12 (25.0)	0/10 (0.0)	0.003
Biological variable, no. (%)							
Baseline CRP level, ≥ 6 mg/L	148	19/85 (22.4)	15/32 (46.9)	5/11 (45.5)	7/10 (70.0)	5/10 (50.0)	0.003
Treatments ^b^, no. (%)							
Steroids and/or IS	185	57/107 (53.3)	33/40 (82.5)	13/13 (100)	12/13 (92.3)	12/12 (100.0)	<.001
Steroids	181	54/108 (50.0)	29/38 (76.3)	11/12 (91.7)	9/12 (75.0)	9/11 (81.8)	0.002
Methotrexate	166	13/103 (12.6)	10/32 (31.3)	3/13 (23.1)	2/9 (22.2)	4/9 (44.4)	0.028
Azathioprine	160	6/100 (6.0)	5/31 (16.1)	2/11 (18.2)	3/9 (33.3)	3/9 (33.3)	0.008
Mycophenolate mofetil	170	21/103 (20.4)	15/34 (44.1)	5/12 (41.7)	8/11 (72.7)	9/10 (90.0)	<.001
Cyclophosphamide	174	18/102 (17.7)	17/36 (47.2)	6/13 (46.2)	8/12 (66.7)	6/11 (54.6)	<.001
Rituximab	153	4/99 (4.0)	0/27 (0.0)	1/11 (9.1)	1/7 (14.3)	1/9 (11.1)	NA

*Anti-RNAP3* anti-RNA polymerase III antibodies, *CRP* C-reactive protein, *disease duration* duration from the first non-RP symptom, *DLCO* diffusing capacity of the lung for carbon monoxide (% of predicted value), *FVC* forced vital capacity (% of predicted value), *IS* immunosuppressive treatment, *NA* not applicable, *RP* Raynaud’s phenomenon, *STPR* skin thickening progression rate

^a^The sum of % may be different from 100% because some patients had either unidentified ANA or multiple autoantibodies

^b^During follow-up

**Fig. 3:**
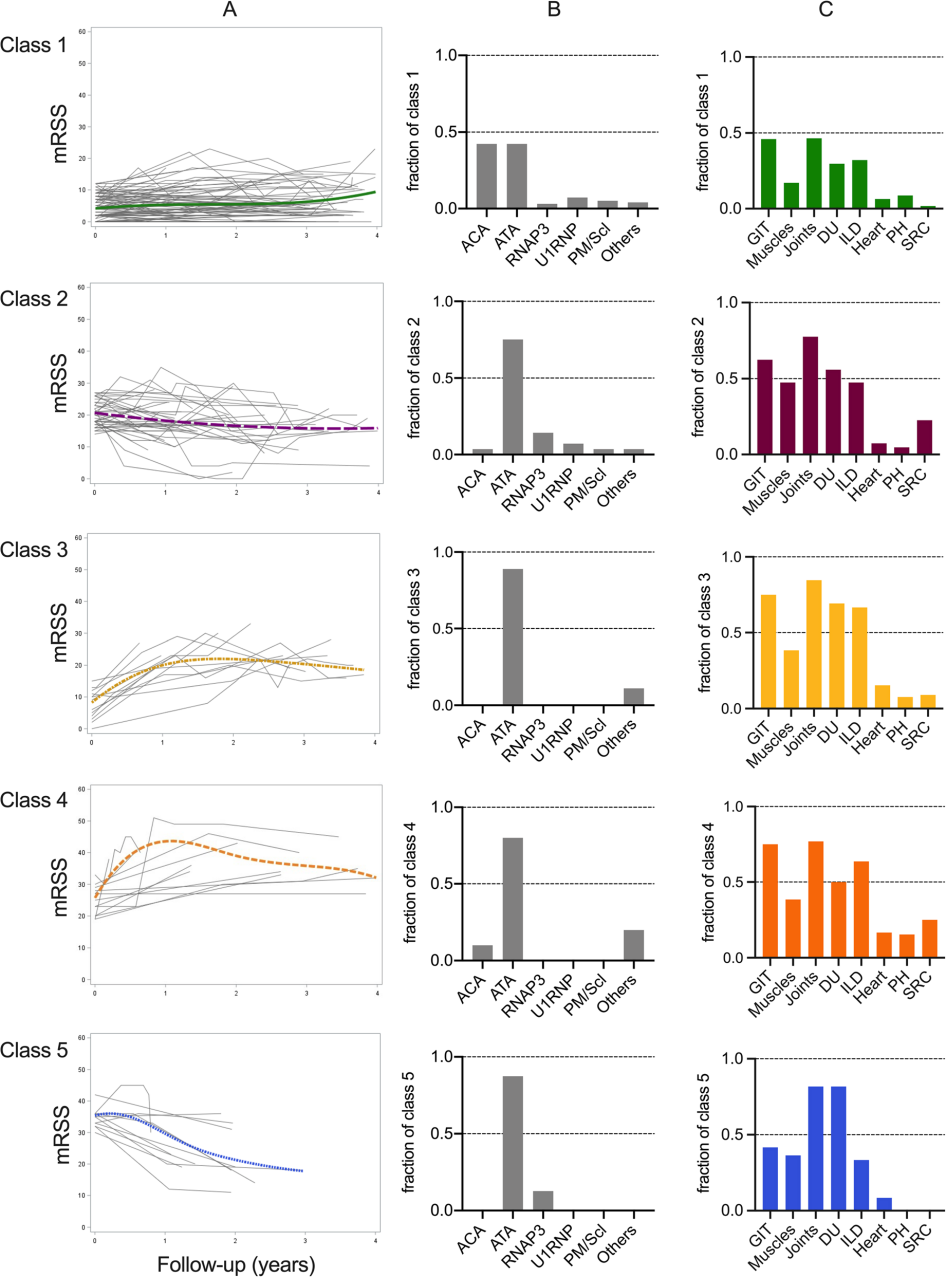
Clinical characteristics of the 5 trajectory classes of the 5-class LCMM. **a** Each class’ spaghetti-plot of the 5-class LCMM with the modeled trajectory estimated using B-splines. Time 0 was defined by the date of the baseline mRSS record. mRSS: modified Rodnan skin score. **b** Graphs representing the autoantibodies in each class. ACA: anti-centromere antibodies; RNAP3: anti-RNA polymerase III antibodies; ATA: anti-topoisomerase I antibodies; others: no specific SSc target antibodies. **c** Graphs illustrating the main organ involvement in each class. DU: digital ulcers; GIT: gastrointestinal tracts; ILD: interstitial lung disease; PH: pulmonary hypertension; SRC: scleroderma renal crisis

*Class 2* slightly improved from a mean baseline mRSS of 20.8 [95% CI 19.0; 22.5] to a mean mRSS at 1 year, 2 years, 3 years, and 4 years of 18.4 [16.3; 20.5], 16.8 [14.5; 19.0], 15.7 [13.6; 17.9], and 15.4 [11.0; 19.8], respectively. This class comprised 43 patients composed of White (87.5%) women (65.1%) with dcSSc (97.6%) associated with ATA (75.0%). Joint, DU, GIT, and ILD involvements were common at baseline. SRC was found in 7 patients (22.6%). The median STPR was 21.8 (IQR 16.0; 35.9) units/year.

*Class 3* was characterized by a 2-step trajectory with a low baseline mRSS (mean mRSS: 8.7 [95% CI 6.0; 11.5]) rapidly increasing to a mean estimated peak mRSS of 23.2 [18.8; 27.6] at 2.3 years of follow-up, then followed by an improvement (mean mRSS at 4 years: 14.5 [8.4; 20.7]). Three of them were Black patients, 11 had dcSSc, and 2 had lcSSc. ATA was common. More than two-thirds of them had joint, DU, GIT, and ILD involvements. The median STPR was 7.8 (IQR 5.7; 13.4) units/year.

*Class 4* was characterized by a 2-step trajectory with a mean baseline mRSS of 25.1 [95% CI 22.6; 27.6], which is rapidly increasing to a mean estimated peak mRSS of 41.6 [37.2; 46.0] at 2.2 years of follow-up, then followed by an improvement (mean mRSS at 4 years: 29.5 [22.7; 36.2]). This class was composed of 13 patients including 6 men and 3 Black patients. ATA, joint, GIT, and ILD involvements were frequent at baseline. The median STPR was 38.5 (IQR: 29.0; 131.3) units/year.

*Class 5* was characterized by a mean baseline mRSS of 35.1 [95% CI 32.2; 37.9] subsequently improving (mean mRSS at 1 year, 2 years, 3 years, and 4 years: 27.6 [23.3; 31.8], 21.5 [17.2; 25.7], 16.8 [10.1; 23.5], and 13.5 [0; 29.5], respectively). All 12 patients had dcSSc mainly associated with ATA. At baseline, most of them had joint and DU involvements. A third was affected by ILD for whom the median FVC and DLCO were 54.0% (IQR: 53.0; 92.0) and 44.0% (41.0; 53.0), respectively (Additional file 10). The median STPR was 34.4 (IQR 27.6; 74.9) units/year.

Steroids and immunosuppressive treatments were more frequently used in classes 2 to 5 than in class 1 (from 82.5% to 100% versus 53.3% in class 1; *p* < .001) during the follow-up. No significant difference was noted between classes 2 to 5 in terms of steroids, methotrexate, azathioprine, cyclophosphamide, and rituximab. Mycophenolate mofetil (MMF) was used more often in class 5 (90.0%) than in classes 2 (44.1%; *p* = 0.013) and 3 (41.7%; *p* = 0.031). No significant difference was found between classes 4 (72.7%) and 5 (90.0%).

### Survival analysis

**Fig. 4:**
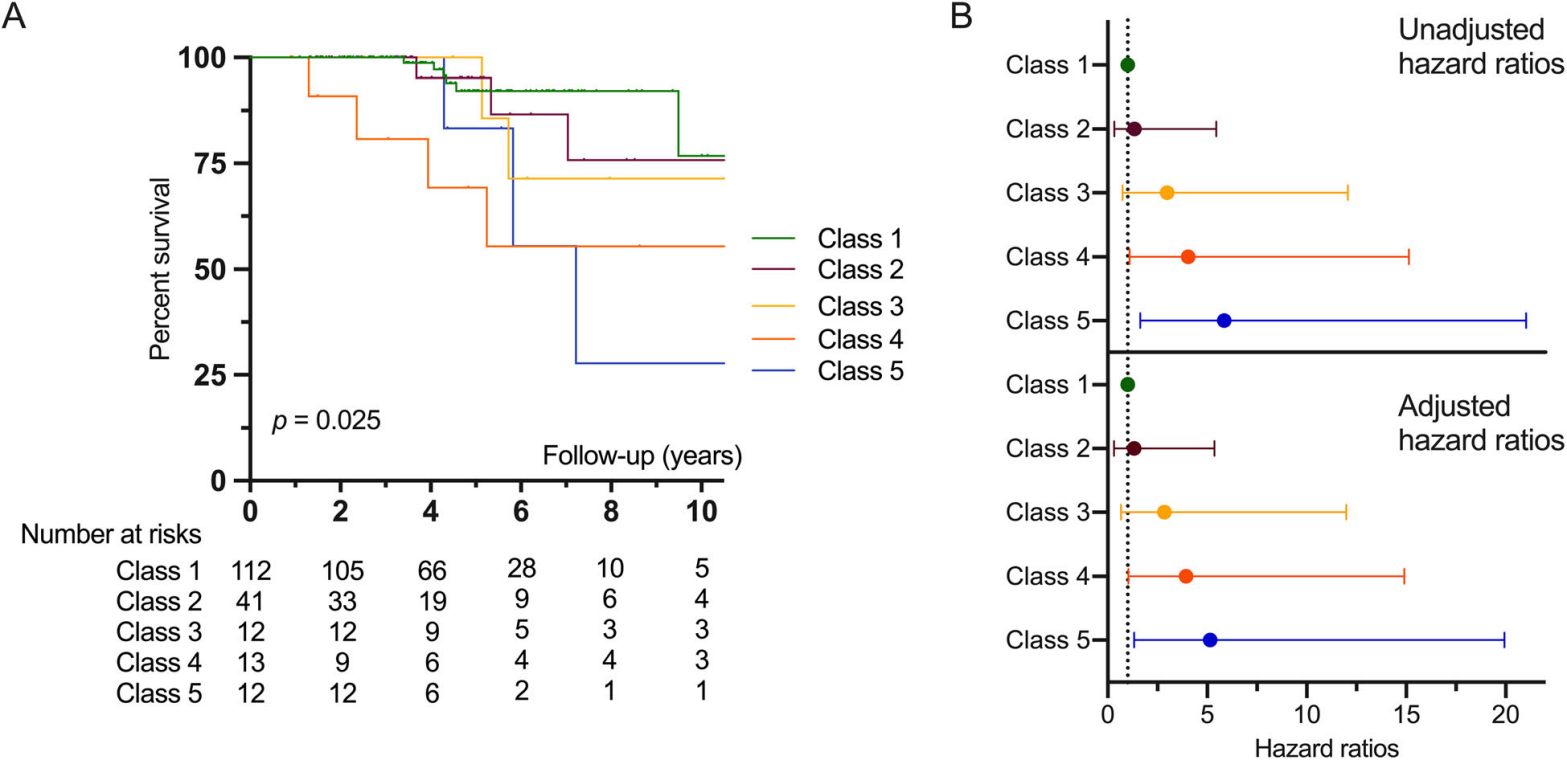
Survival of the 5 trajectory classes of the 5-class LCMM*.* **a** Kaplan-Meier curves of 5-class LCMM (*p* = 0.025). Time 0 was defined by the date of the first non-Raynaud’s Phenomenon symptom. **b** Forest plot showing mortality hazard ratios and 95% confidence intervals for the 5 trajectory classes before (top) or after (bottom) adjusting for sex and age. Broken line shows the hazard ratio for the reference group

Kaplan-Meier curves are shown in Fig. 4. Survival was different according to the trajectory classes (*p* = 0.025). Using Cox regression analysis (Additional file 11), we observed a progressive increase in the risk of death from classes 2 to 5 compared with class 1 (reference): hazard ratio (HR) (class 2) = 1.35 [95% CI 0.33; 5.46], HR (class 3) = 2.99 [0.74; 12.07], HR (class 4) = 4.05 [1.09; 15.13], and HR (class 5) = 5.85 [1.63; 21.03]. The results were similar after adjusting for age and sex (Fig. 4, Additional file 12).

## Discussion

The main results were as follows: (i) LCMM identified without any a priori assumptions five distinct trajectories of mRSS during the follow-up in early SSc patients under standard care (inclusion within 2 years of the first non-RP symptom), (ii) the mRSS trajectory classes were associated with different organ involvement and survival, and (iii) patients with high baseline mRSS or those who peaked after baseline had the highest severity.

The natural evolution of skin thickening is very heterogeneous, yet it is generally accepted that it tends to worsen at the beginning of dcSSc to a maximum, which usually occurs over the first 2–3 years after disease onset, then followed by improvement at the advanced stage [10, 21–23]. However, the data related to this are limited and complex. Modeling the evolution of mRSS has only been performed in a few studies. Shand et al. [6] classified 131/192 (68%) early dcSSc patients into 3 subgroups using latent trajectory modeling over the first 2 years of follow-up. Those three subgroups, “low baseline (mean mRSS: 20 ± 6)/improvers,” “high baseline (mean mRSS: 42 ± 8)/non-improvers,” and “high baseline (mean mRSS 35 ± 7)/improvers,” had similar trajectories than classes 2, 4, and 5. The survival rate in the “high baseline/non-improvers” (similar to the class 4 in our study) subgroup was significantly worse than that in the 2 other subgroups. In our study, we observed that classes 4 and 5 had the worst survival. Moreover, we found 2 additional trajectories: one mainly composed of lcSSc (class 1), and another one characterized by a 2-step trajectory with a low baseline mRSS rapidly increasing to a mean estimated peak mRSS of 23.2 [95% CI 18.8; 27.6] before improving (class 3). We may have captured this last trajectory due to the following reasons: (i) we included patients with lcSSc, and (ii) the very short disease duration and the longer follow-up (4 years) in our study allowed us to discriminate these individual trajectories with different patterns of early skin change. Using a prespecified definition of progressive skin disease (increase in mRSS of > 5 points and ≥ 25% from baseline), Maurer et al. [9] identified several independent factors associated with skin thickening progression: baseline mRSS of ≤ 22/51, low baseline STPR, and disease duration of ≤ 15 months. The best prediction model of worsening performed correctly in only 44.4% of the cases, suggesting that it is still not easy to accurately predict the skin thickening progression. To our knowledge, no study has attempted to model the evolution of mRSS without any a priori assumptions or prespecified definition.

Our approach identified five original distinct trajectories over time meeting the fittest formal statistical criteria, model adequacy, and clinical relevance of discriminated trajectories. These five classes of trajectories were distinguished by intrinsic characteristics such as baseline mRSS, trajectory slopes of worsening or improvement, and mRSS peaks. The 5-class model remained the best model to decipher the global heterogeneity with disease duration as an adjustment factor. Class 1 had low mRSS and STPR values at baseline, less of organ involvements at baseline, and better survival, as reported in lcSSc [24–26]. Most patients with ACA (95%) were assigned to class 1. However, 42% of patients in class 1 had ATA, which is a higher proportion than that usually observed in lcSSc [24, 27, 28]. In addition to lcSSc, there were 20 patients (17%) with dcSSc associated with ATA and whose median baseline mRSS was 9 (IQR 6.5; 9.5). Low mRSS values have already been reported in dcSSc [29–31] and some limitations on the current classification have been identified [28, 32], especially when the forearms are involved. Their assignation to class 1 was probably related to the modeled trajectory shape of class 1 that was fitter to their individual trajectories than the other modeled trajectories classes.

Organ involvement was more frequent in classes 2 to 5 than in class 1. As expected, the use of immunosuppressive drugs was also more frequent in classes 2 to 5 than in class 1. With regard to survival, we found an increasing risk of death from classes 2 to 5 compared with class 1, especially in trajectory classes with a high baseline mRSS (class 5) or an intermediate baseline mRSS that peaked after baseline (class 4). These 2 classes also shared a rapid STPR at baseline (median > 30 units/years) compared with other classes. In our study, patients with ATA were present in all five trajectory classes while we noted that lcSSc patients were predominantly in class 1 and those with dcSSc were mainly in classes 2 to 5. This heterogeneity is well known and has been confirmed by several recent works highlighting the importance of autoantibodies, cutaneous subset, and disease duration to predict organ complications and survival [7, 23, 28]. In particular, it appears relevant to distinguish especially ATA-associated lcSSc from ATA-associated dcSSc [33, 34]. Recently, Wu et al. reported that in dcSSc, skin progression that occurred within 1 year was independently associated with forced vital capacity decline and all-cause death [35], and Zheng et al. reported worst disease outcomes in early dcSSc patients with worsening skin score (≤ 3 years), whose data were extracted from the Canadian Scleroderma Research Group database [36]. Taken together, these results highlight the need to consider the baseline mRSS and the early changes in skin thickening (worsening/improving) in patient risk stratification.

Our study has the following strengths: we used prospective data from a French multicenter cohort on SSc including patients with early disease duration from the first non-RP symptom (≤ 2 years). We were able to identify five trajectory classes using an approach without any a priori assumptions. Moreover, the mRSS changes that occurred in the trajectory classes 2 to 5 seemed clinically relevant. Indeed, they were higher than the minimal clinically important difference in mRSS (≥ 3–4 units) estimated in the Scleroderma Lung Studies [37]. Finally, we showed under standard care that patients with high baseline mRSS or those whose mRSS peaked after baseline had the highest severity.

The potential limitations include firstly potential biases in relation to inclusion criteria. Skin involvement, which is an important outcome in dcSSc, could have been more frequently assessed in dcSSc patients explaining a higher proportion of men with dcSSc, ATA, and anti-RNAP3 among included patients than among excluded patients. However, mortality did not seem different between the two groups (Additional file 1). We cannot exclude having missed out the most severe patients who would have died before the second record. Second, we chose to include patients with a disease duration of ≤ 2 years. A longer disease duration might have enabled a larger study size but would have increased the proportion of patients in which the mRSS has already been reached (usually during the first 2–3 years of the disease) [29]. A shorter disease duration could also have been more relevant but would have reduced the sample size preventing a robust modeling. Third, the disease duration appeared longer in class 5 compared to class 4, but modeling with disease duration as adjustment factor confirmed that the 5-class model outperformed the other models, suggesting that the 5-class model remained as the optimal model. The small size of some classes (reflecting that patients assigned to them were rarely encountered in clinical practice) and the unknown causes of death may limit the interpretation of data. Fourth, mRSS was not recorded by the same practitioner for each patient leading to potential inter-observer variability. The long duration of the study and the turnover of the investigators could have led to a high variability in mRSS. However, all participating centers are SSc referral centers in which physicians attend regular mRSS assessment training, which might have reduced this limitation [38, 39]. Fifth, the start and end dates for each immunosuppressive drug in parallel to mRSS assessments were lacking in the database. Therefore, the influence of immunosuppression could not be considered for the trajectory modeling. Finally, our findings should be confirmed on an external validation cohort.

## Conclusion

This study identified five distinct mRSS trajectories in early SSc patients. The mRSS trajectory classes were associated with organ involvement and survival. Early identification of clinical phenotype based on skin thickening trajectories could predict morbi-mortality in SSc and influence clinical management.

## Supplementary information

**Additional file 1. **Demographics and disease characteristics of patients with less than 2 years of the first non-RP symptom (*n* = 611)

**Additional file 2.** Number of mRSS available in patients included in LCMM.

**Additional file 3.** The 6 different LCMM tested

**Additional file 4.** Model fit evaluation information for each LCMM tested

**Additional file 5.** Averages of posterior probabilities of belonging to a class in each LCMM tested

**Additional file 6.** Averages of posterior probabilities of belonging to a class in the 5-class LCMM

**Additional file 7.** Sensitivity analysis: the 6 different LCMM with disease duration as adjustment factor

**Additional file 8.** Sensitivity analysis: model fit evaluation information for each LCMM with disease duration as adjustment factor

**Additional file 9.** Sensitivity analysis: averages of posterior probabilities of belonging to a class in each LCMM with disease duration as adjustment factor

**Additional file 10. **Pulmonary function test values in patients with interstitial lung disease in the 5-class model (*n* = 72)

**Additional file 11.** Survival analyses using Cox regression analysis without adjustment for age and sex in the 5-class LCMM

**Additional file 12.** Survival analyses using Cox regression analysis adjusted for age and sex in the 5-class LCMM

## Data Availability

The data that support the findings of this study are available on separate scientific request (contact Prof. Eric Hachulla, Department of Internal Medicine, University of Lille, Lille, France; eric.hachulla@chru-lille.fr), but restrictions apply to the availability of these data, which were used under license for the current study and, hence, are not publicly available.

## Abbreviations

ACA: Anti-centromere antibodies
Anti-RNAP3: Anti-RNA polymerase III antibodies
ATA: Anti-topoisomerase I antibodies
BIC: Bayes information criterion
dcSSc: Diffuse systemic sclerosis
DLCO: Diffusing capacity of the lung for carbon monoxide (% predicted value)
DU: Digital ulcers
FVC: Forced vital capacity (% predicted value)
GIT: Gastrointestinal tract
ILD: Interstitial lung disease
IQR: Interquartile range
LCMM: Latent class mixed model
lcSSc: Limited cutaneous systemic sclerosis
MMF: Mycophenolate mofetil
mRSS: Modified Rodnan skin score
PH: Pulmonary hypertension
RP: Raynaud’s phenomena
SD: Standard deviation
SRC: Scleroderma renal crisis
SSc: Systemic sclerosis
STPR: Skin thickening progression rate
