# Supplementary material for: Early trajectories of skin thickening are associated with severity and mortality in systemic sclerosis
Source: Arthritis Res Ther. 2020 Feb 18;22:30. doi: 10.1186/s13075-020-2113-6 (PMC7029583; doi:10.1186/s13075-020-2113-6)
Supplement: Supplementary file 1 — Additional file 1. Demographics and disease characteristics of patients with less than 2 years of the first non-RP symptom (n = 611) [file 13075_2020_2113_MOESM1_ESM.docx]

**Additional file 1.** Demographics and disease characteristics of patients with less than 2 years of the first non-RP symptom (n = 611)

|  | **No. with available data** | **≤ 1 mRSS available**  **(n = 413)** | **No. with available data** | **Patients included**  **(n = 198)** | **p** |
| --- | --- | --- | --- | --- | --- |
| **Demographics** |  |  |  |  |  |
| Sex, female, no. (%) | 413 | 336 (81.4) | 198 | 145 (73.2) | 0.022 |
| Ethnicity, no. (%) |  |  |  |  |  |
| White | 315 | 286 (90.8) | 161 | 140 (87.0) | 0.43 |
| Black | 315 | 22 (7.0) | 161 | 16 (9.9) |  |
| Asian | 315 | 7 (2.2) | 161 | 5 (3.1) |  |
| Age, median (±SD), y | 413 | 52.3 ±15.8 | 198 | 51.1 ±14.3 | 0.39 |
| **Disease characteristics** |  |  |  |  |  |
| Autoantibody status ^a^, no. (%) |  |  |  |  |  |
| Anti-nuclear antibody positive | 300 | 281 (93.7) | 165 | 156 (94.5) | 0.70 |
| Anti-centromere | 280 | 153 (54.6) | 152 | 43 (28.3) | <.001 |
| Anti-topoisomerase I | 280 | 97 (34.6) | 152 | 85 (55.9) | <.001 |
| Anti-RNAP3 | 280 | 3 (1.1) | 152 | 8 (5.3) | 0.020 |
| Anti-U1 RNP | 280 | 17 (6.1) | 152 | 9 (5.9) | 0.95 |
| Anti-PM/Scl | 280 | 11 (3.9) | 152 | 6 (4.0) | >.99 |
| Disease duration, median (IQR), y | 413 | 0.7 (0.3; 1.2) | 198 | 0.8 (0.4; 1.2) | 0.018 |
| Duration from RP, median (IQR), y | 371 | 2.0 (0.7; 6.3) | 187 | 1.3 (0.6; 3.9) | 0.004 |
| Follow up, median (IQR), y | 310 | 9.7 (5.0; 15.0) | 198 | 6.3 (3.8; 8.9) | <.001 |
| Cutaneous subset, limited, no. (%) | 400 | 292 (73.0) | 195 | 98 (50.3) | <.001 |
| **Baseline organ involvement,**  **no. (%)** |  |  |  |  |  |
| Telangiectasia | 367 | 168 (45.8) | 183 | 76 (41.5) | 0.35 |
| Calcinosis | 365 | 41 (11.2) | 175 | 20 (11.4) | 0.95 |
| Joints | 387 | 207 (53.5) | 191 | 114 (59.7) | 0.16 |
| Muscles | 401 | 104 (25.9) | 194 | 53 (27.3) | 0.72 |
| Digital ulcers | 322 | 135 (41.9) | 181 | 76 (42.0) | >.99 |
| Gastrointestinal tracts | 379 | 135 (35.6) | 187 | 99 (52.9) | <.001 |
| Interstitial lung disease | 368 | 131 (35.6) | 181 | 72 (39.8) | 0.34 |
| FVC, median % (IQR) | 269 | 96.0 (74.0; 110.0) | 160 | 96.0 (76.0; 108.0) | >.99 |
| DLCO, median % (IQR) | 274 | 69.0 (55.0; 81.0) | 155 | 63.0 (50.0; 78.0) | 0.024 |
| Heart | 377 | 37 (9.8) | 187 | 15 (8.0) | 0.49 |
| Pulmonary hypertension | 388 | 31 (8.0) | 194 | 15 (7.7) | 0.91 |
| Renal crisis | 300 | 42 (14.0) | 123 | 12 (9.8) | 0.23 |
| **Biological variables, no. (%)** |  |  |  |  |  |
| Baseline CRP level, ≥6mg/L | 278 | 89 (32.0) | 148 | 51 (34.5) | 0.61 |
| **Treatments ^b^, no. (%)** |  |  |  |  |  |
| Steroids and/or IS | 296 | 189 (63.9) | 189 | 127 (68.7) | 0.28 |
| Steroids | 328 | 169 (51.5) | 181 | 112 (61.9) |  |
| Methotrexate | 283 | 43 (15.2) | 166 | 32 (19.3) |  |
| Azathioprine | 276 | 21 (7.6) | 160 | 19 (11.8) |  |
| Mycophenolate mofetil | 272 | 39 (14.3) | 170 | 58 (34.1) |  |
| Cyclophosphamide | 285 | 57 (20.0) | 174 | 55 (31.6) |  |
| Rituximab | 184 | 5 (2.7) | 153 | 7 (4.6) |  |

Numbers are given as % or mean ±standard deviation (SD) or median with interquartile range (IQR). Anti-RNAP3: anti-RNA polymerase III antibodies; CRP: C-reactive protein; disease duration: duration from the first non-RP symptom; DLCO: diffusing capacity of the lung for carbon monoxide (% of predicted value); FVC: forced vital capacity (% of predicted value); RP: Raynaud’s phenomenon; y: years; ^a^the sum of % may be different from 100% because some patients had either unidentified ANA or multiple autoantibodies; ^b^during follow up
