# Supplementary material for: Early trajectories of skin thickening are associated with severity and mortality in systemic sclerosis
Source: Arthritis Res Ther. 2020 Feb 18;22:30. doi: 10.1186/s13075-020-2113-6 (PMC7029583; doi:10.1186/s13075-020-2113-6)
Supplement: Supplementary file 2 — Additional file 2. Number of mRSS available in patients included in LCMM. [file 13075_2020_2113_MOESM2_ESM.docx]

**Additional file 2.** Number of mRSS available in patients included in LCMM

| **mRSS recorded** | **Frequency** | **Cumulative percent (%)** |
| --- | --- | --- |
| 2 | 89 | 45.0 |
| 3 | 45 | 67.7 |
| 4 | 32 | 83.9 |
| 5 | 14 | 90.9 |
| 6 | 8 | 95.0 |
| 7 | 5 | 97.5 |
| 8 | 1 | 98.0 |
| 9 | 2 | 99.0 |
| 10 | 1 | 99.5 |
| 11 | 1 | 100 |

mRSS: modified Rodnan skin score
