# Supplementary material for: Early trajectories of skin thickening are associated with severity and mortality in systemic sclerosis
Source: Arthritis Res Ther. 2020 Feb 18;22:30. doi: 10.1186/s13075-020-2113-6 (PMC7029583; doi:10.1186/s13075-020-2113-6)
Supplement: Supplementary file 3 — Additional file 3. The 6 different LCMM tested [file 13075_2020_2113_MOESM3_ESM.docx]

**Additional file 3.** The 6 different LCMM tested

**

**Number of patients: 198. (**A)** 1-class LCMM. (**B)** 2-class LCMM. (**C)** 3-class LCMM. (**D)** 4-class LCMM. (**E)** 5-class LCMM. (**F)** 6-class LCMM. LCMM: latent class mixed model. Time 0 was defined by the date of baseline mRSS record
