# Supplementary material for: Early trajectories of skin thickening are associated with severity and mortality in systemic sclerosis
Source: Arthritis Res Ther. 2020 Feb 18;22:30. doi: 10.1186/s13075-020-2113-6 (PMC7029583; doi:10.1186/s13075-020-2113-6)
Supplement: Supplementary file 4 — Additional file 4. Model fit evaluation information for each LCMM tested [file 13075_2020_2113_MOESM4_ESM.docx]

**Additional file 4.** Model fit evaluation information for each LCMM tested

|  | **Maximum log-likelihood** | **% reduction in**  **log-likelihood from the previous model** | **Likelihood**  **ratio test**  *p***-value** | **AIC** | **BIC** |
| --- | --- | --- | --- | --- | --- |
| One-class LCMM | -2170.0 | - | - | 4360 | 4393 |
| Two-class LCMM | -2138.3 | 1.47 | <.001 | 4305 | 4351 |
| Three-class LCMM | -2115.6 | 1.06 | <.001 | 4267 | 4326 |
| Four-class LCMM | -2106.2 | 0.44 | 0.053 | 4256 | 4329 |
| Five-class LCMM | -2088.2 | 0.85 | <.001 | 4228 | 4314 |
| Six-class LCMM | -2084.4 | 0.18 | 0.44 | 4229 | 4328 |

AIC: Akaike information criteria; BIC: Bayesian information criteria; LCMM: latent class mixed model
