# Supplementary material for: Early trajectories of skin thickening are associated with severity and mortality in systemic sclerosis
Source: Arthritis Res Ther. 2020 Feb 18;22:30. doi: 10.1186/s13075-020-2113-6 (PMC7029583; doi:10.1186/s13075-020-2113-6)
Supplement: Supplementary file 5 — Additional file 5. Averages of posterior probabilities of belonging to a class in each LCMM tested [file 13075_2020_2113_MOESM5_ESM.docx]

**Additional file 5.** Averages of posterior probabilities of belonging to a class in each LCMM tested

|  | **Class 1** | **Class 2** | **Class 3** | **Class 4** | **Class 5** | **Class 6** |
| --- | --- | --- | --- | --- | --- | --- |
| One-class LCMM | - | - | - | - | - | - |
| Two-class LCMM | 0.982 | 0.922 | - | - | - | - |
| Three-class LCMM | 0.891 | 0.968 | 0.841 | - | - | - |
| Four-class LCMM | 0.965 | 0.867 | 0.884 | 0.780 | - | - |
| Five-class LCMM | 0.960 | 0.881 | 0.922 | 0.954 | 0.930 | - |
| Six-class LCMM | 0.952 | 0.955 | 0.914 | 0.878 | 0.878 | 0.632 |

LCMM: latent class mixed model
