# Supplementary material for: Early trajectories of skin thickening are associated with severity and mortality in systemic sclerosis
Source: Arthritis Res Ther. 2020 Feb 18;22:30. doi: 10.1186/s13075-020-2113-6 (PMC7029583; doi:10.1186/s13075-020-2113-6)
Supplement: Supplementary file 6 — Additional file 6. Averages of posterior probabilities of belonging to a class in the 5-class LCMM [file 13075_2020_2113_MOESM6_ESM.docx]

**Additional file 6.** Averages of posterior probabilities of belonging to a class in the 5-class LCMM

|  | **Prob. A** | **Prob. B** | **Prob. C** | **Prob. D** | **Prob. E** |
| --- | --- | --- | --- | --- | --- |
| Class 1 | **0.960** | 0.008 | 0.032 | 0.000 | 0.000 |
| Class 2 | 0.027 | **0.881** | 0.035 | 0.039 | 0.018 |
| Class 3 | 0.050 | 0.028 | **0.922** | 0.001 | 0.000 |
| Class 4 | 0.000 | 0.033 | 0.011 | **0.954** | 0.002 |
| Class 5 | 0.000 | 0.060 | 0.000 | 0.012 | **0.930** |

LCMM: latent class mixed model
