# Supplementary material for: Early trajectories of skin thickening are associated with severity and mortality in systemic sclerosis
Source: Arthritis Res Ther. 2020 Feb 18;22:30. doi: 10.1186/s13075-020-2113-6 (PMC7029583; doi:10.1186/s13075-020-2113-6)
Supplement: Supplementary file 8 — Additional file 8. Sensitivity analysis: model fit evaluation information for each LCMM with disease duration as adjustment factor [file 13075_2020_2113_MOESM8_ESM.docx]

**Additional file 8.** Sensitivity analysis: model fit evaluation information for each LCMM with disease duration as adjustment factor

|  | **Maximum log-likelihood** | **% reduction in**  **log-likelihood from the previous model** | **Likelihood**  **ratio test**  *p***-value** | **AIC** | **BIC** |
| --- | --- | --- | --- | --- | --- |
| One-class LCMM | -2169.08 | - | - | 4360.16 | 4396.33 |
| Two-class LCMM | -2136.43 | 1.51 | <.001 | 4306.86 | 4362.76 |
| Three-class LCMM | -2110.36 | 1.22 | <.001 | 4266.73 | 4342.36 |
| Four-class LCMM | -2100.66 | 0.46 | 0.14 | 4259.31 | 4354.67 |
| Five-class LCMM | -2080.24 | 0.98 | 0.002 | 4230.47 | 4345.56 |
| Six-class LCMM | -2070.92 | 0.44 | 0.16 | 4223.83 | 4358.65 |

AIC: Akaike information criteria; BIC: Bayesian information criteria; LCMM: latent class mixed model
