# Supplementary material for: Early trajectories of skin thickening are associated with severity and mortality in systemic sclerosis
Source: Arthritis Res Ther. 2020 Feb 18;22:30. doi: 10.1186/s13075-020-2113-6 (PMC7029583; doi:10.1186/s13075-020-2113-6)
Supplement: Supplementary file 9 — Additional file 9. Sensitivity analysis: averages of posterior probabilities of belonging to a class in each LCMM with disease duration as adjustment factor [file 13075_2020_2113_MOESM9_ESM.docx]

**Additional file 9.** Sensitivity analysis: averages of posterior probabilities of belonging to a class in each LCMM with disease duration as adjustment factor

|  | **Class 1** | **Class 2** | **Class 3** | **Class 4** | **Class 5** | **Class 6** |
| --- | --- | --- | --- | --- | --- | --- |
| One-class LCMM | - | - | - | - | - | - |
| Two-class LCMM | 0.981 | 0.935 | - | - | - | - |
| Three-class LCMM | 0.847 | 0.966 | 0.898 | - | - | - |
| Four-class LCMM | 0.954 | 0.853 | 0.912 | 0.849 | - | - |
| Five-class LCMM | 0.965 | 0.956 | 0.935 | 0.868 | 0.897 | - |
| Six-class LCMM | 0.931 | 0.968 | 0.928 | 0.928 | 0.855 | 0.772 |

LCMM: latent class mixed model
