# Supplementary material for: Early trajectories of skin thickening are associated with severity and mortality in systemic sclerosis
Source: Arthritis Res Ther. 2020 Feb 18;22:30. doi: 10.1186/s13075-020-2113-6 (PMC7029583; doi:10.1186/s13075-020-2113-6)
Supplement: Supplementary file 10 — Additional file 10. Pulmonary function test values in patients with interstitial lung disease in the 5-class model (n = 72) [file 13075_2020_2113_MOESM10_ESM.docx]

**Additional file 10.** Pulmonary function test values in patients with interstitial lung disease in the 5-class model (n = 72)

|  | **No. with available data** | **Class 1**  **(n = 117)** | **Class 2**  **(n = 43)** | **Class 3**  **(n = 13)** | **Class 4**  **(n = 13)** | **Class 5**  **(n = 12)** | **p** |
| --- | --- | --- | --- | --- | --- | --- | --- |
| Interstitial lung disease ^a^ | 181 | 34/106 (32.1) | 19/40 (47.5) | 8/12 (66.7) | 7/11 (63.6) | 4/12 (33.3) | 0.040 |
| FVC, median % (IQR) ^b^ | 63/72 | 88 (64; 103) | 84 (63; 86) | 75 (66; 108) | 78 (58; 93) | 54 (53; 92) | NA |
| DLCO, median % (IQR) ^b^ | 60/72 | 52 (36; 69) | 54 (46; 61) | 59 (50; 83) | 58 (37; 83) | 44 (41; 53) | NA |

DLCO: diffusing capacity of the lung for carbon monoxide (% predicted value); FVC: forced vital capacity (% predicted value); NA: not applicable; ^a^interstitial lung disease on HRCT or chest X-ray; ^b^among patients with interstitial lung disease
