# Supplementary material for: Early trajectories of skin thickening are associated with severity and mortality in systemic sclerosis
Source: Arthritis Res Ther. 2020 Feb 18;22:30. doi: 10.1186/s13075-020-2113-6 (PMC7029583; doi:10.1186/s13075-020-2113-6)
Supplement: Supplementary file 11 — Additional file 11. Survival analyses using Cox regression analysis without adjustment for age and sex in the 5-class LCMM [file 13075_2020_2113_MOESM11_ESM.docx]

**Additional file 11.** Survival analyses using Cox regression analysis without adjustment for age and sex in the 5-class LCMM

|  | **Hazard ratio** | **95% CI** | **p-value** |
| --- | --- | --- | --- |
| Class 1 | Reference | Reference | Reference |
| Class 2 | 1.35 | [0.33; 5.46] | 0.67 |
| Class 3 | 2.99 | [0.74; 12.07] | 0.12 |
| Class 4 | 4.05 | [1.09; 15.13] | 0.037 |
| Class 5 | 5.85 | [1.63; 21.03] | 0.007 |
