# Supplementary material for: Early trajectories of skin thickening are associated with severity and mortality in systemic sclerosis
Source: Arthritis Res Ther. 2020 Feb 18;22:30. doi: 10.1186/s13075-020-2113-6 (PMC7029583; doi:10.1186/s13075-020-2113-6)
Supplement: Supplementary file 12 — Additional file 12. Survival analyses using Cox regression analysis adjusted for age and sex in the 5-class LCMM [file 13075_2020_2113_MOESM12_ESM.docx]

**Additional file 12.** Survival analyses using Cox regression analysis adjusted for age and sex in the 5-class LCMM

|  | **Hazard ratio** | **95% CI** | **p-value** |
| --- | --- | --- | --- |
| Class 1 | Reference | Reference | Reference |
| Class 2 | 1.32 | [0.32; 5.36] | 0.70 |
| Class 3 | 2.84 | [0.67; 11.99] | 0.16 |
| Class 4 | 3.93 | [1.04; 14.90] | 0.044 |
| Class 5 | 5.14 | [1.32; 19.94] | 0.018 |
